# 5-Bromoprotocatechualdehyde Combats against Palmitate Toxicity by Inhibiting Parkin Degradation and Reducing ROS-Induced Mitochondrial Damage in Pancreatic β-Cells

**DOI:** 10.3390/antiox10020264

**Published:** 2021-02-09

**Authors:** Seon-Heui Cha, Chunying Zhang, Soo-Jin Heo, Hee-Sook Jun

**Affiliations:** 1 Department of Marine Bio and Medical Sciences, Hanseo University, Chungcheongman-do 31962, Korea; 2 Department of Integrated of Bioindustry, Hanseo University, Chungcheongman-do 31962, Korea; zhangchunying1986@yeah.net; 3 Department of Biology, University of Science and Technology (UST), Daejeon 34113, Korea; 4 Marine Research Center, Institute of Ocean Science and Technology (KIOST), Jeju 63349, Korea; 5 Lee Gil Ya Cancer and Diabetes Institute, Gachon University, Incheon 21999, Korea; 6 Gachon Medical and Convergence Institute, Gachon Gil Medical Center, Incheon 21565, Korea; 7 Department of Pharmacology, Gachon University, Incheon 21936, Korea

**Keywords:** β-cell, diabetes, seaweed, *Polysiphonia japonica*, polyphenol, 5-bromoprotocatechualdehyde

## Abstract

Pancreatic β-cell loss is critical in diabetes pathogenesis. Up to now, no effective treatment has become available for β-cell loss. A polyphenol recently isolated from *Polysiphonia japonica*, 5-Bromoprotocatechualdehyde (BPCA), is considered as a potential compound for the protection of β-cells. In this study, we examined palmitate (PA)-induced lipotoxicity in Ins-1 cells to test the protective effects of BPCA on insulin-secreting β-cells. Our results demonstrated that BPCA can protect β-cells from PA-induced lipotoxicity by reducing cellular damage, preventing reactive oxygen species (ROS) overproduction, and enhancing glucose-stimulated insulin secretion (GSIS). BPCA also improved mitochondrial morphology by preserving parkin protein expression. Moreover, BPCA exhibited a protective effect against PA-induced β-cell dysfunction in vivo in a zebrafish model. Our results provide strong evidence that BPCA could be a potential therapeutic agent for the management of diabetes.

## 1. Introduction

Diabetes mellitus (DM) is a group of chronic metabolic disorders diagnosed by high levels of blood sugar over a prolonged period of time and a consequent insulin deficiency. DM has become one of the most concerning diseases due to its association with several complications [1,2,3] and the rise in its global incidence (International Diabetes Federal, IDF diabetes atlas, seventh edition). Although the three major types of DM, i.e., type 1 diabetes (T1D), type 2 diabetes (T2D), and gestational diabetes, have different etiologies, they all feature a crucial pathological transition into hyperglycemia and a consequent overproduction of reactive oxygen species (ROS) [4]. ROS constitute a heterogeneous group of highly reactive molecules that includes free radicals, such as superoxide radicals, hydroxyl radicals, peroxyl radicals, and hydroperoxyl radicals, as well as non-radical species, such as hydrogen peroxide and hydrochloric acid [5,6].

Pancreatic β-cells are essential for the maintenance of glucose homeostasis and function by sensing the elevated blood glucose levels and subsequently producing the glucose-lowering hormone insulin. Sufficient pancreatic β-cell mass and reserves are achieved through β-cell proliferation, which has a critical impact on long-term prevention of T2D [7]. Moreover, under pathologic cases, such as insulin resistance in T2D, β-cell proliferation is enhanced in response to increased insulin demand [8]. It is well known that β-cell proliferation is to increase β-cell mass right before the loss of β-cells to control hyperglycemia in advanced diabetes. [8,9]. Thus, a strict regulation of β-cell mass through cell replication, regulation of cellular size, and apoptotic elimination of particular groups of cells under physiological and pathophysiological conditions is of critical importance. T2D has strong links with obesity and is characterized by high glucose levels, which are often accompanied by excessive amounts of fatty acids, such as palmitate, which is lipotoxic. Abnormal islet function can lead to increased lipid esterification and elevated levels of oxidative stress markers in patients due to redox imbalance caused by overproduction of ROS and reactive nitrogen species (RNS) [10,11,12]. Furthermore, high oxygen consumption is associated with insulin secretion, particularly when the levels of blood glucose are elevated [13], and can lead to increased ROS levels and oxidative stress in β-cells. Due to the insufficient antioxidant enzymes, β-cells are vulnerable to oxidative stress. Therefore, maintaining β-cell health and preventing β-cell degeneration are essential approaches in prevention and/or treatment of DM.

The mitochondria are crucial centers in pancreatic β-cells, as they couple glucose metabolism with insulin exocytosis to ensure a strict control for glucose-stimulated insulin secretion. Accordingly, mitochondrial dysfunction impairs this metabolic coupling and results in β-cell death via apoptosis [14]. The antiapoptotic (Bcl-2, Bcl-xL, Bcl-w, Mcl-1, and A1/Bfl1) and proapoptotic (Bax, Bak, and Bok/Mtd) members of the Bcl-2 family are the major regulators determining the fate of β-cells, and their complex interactions and balance regulate apoptosis by controlling mitochondrial cell death signals, which can be induced by lipotoxicity or pro-inflammatory cytokines [15,16]. In T2D, the stimulation of β-cell apoptotic pathways is mainly due to free fatty acids (FFAs, such as palmitic acid (PA)) and high glucose, which is known as glucolipotoxicity [17]. Exposure of β-cells to glucolipotoxic molecules or pro-inflammatory cytokines induces abnormal signaling pathways, such as Bax and caspases, to initiate apoptosis [18,19,20]. The parkin protein is known to play an important role in regulating mitochondrial function. Recently, it has been reported that parkin is involved in the production and secretion of insulin, a function of pancreatic β-cells [21]; in addition, when the free fatty acid was overintroduced in hhperglycemic condition, the parkin protein expression in insulin-secreting cells was reduced [22].

Phytochemicals have been used for treatment of several health problems, including DM. Numerous studies have reported the beneficial effects of phytotherapy, including the use of seaweeds, on diabetes [23,24,25]. Seaweeds contain a variety of bioactive substances, including polyphenols, polysaccharides, pigments, minerals, and peptides, with valuable pharmaceutical and biomedical potential [26,27,28,29,30,31]. Polyphenols can act as antioxidants against oxidative stress and damage; accordingly, they are relevant for DM [32]. Moreover, polyphenols help prevent diabetes by inhibiting glucose absorption in the intestine, increasing insulin secretion in the pancreas, improving glucose absorption in muscle and adipose cells, and inhibiting glucose release from the liver [33]. In the present study, we isolated the polyphenolic compound 5-bromoprotocatechualdehyde (BPCA) from *Polysiphonia japonica*, a red seaweed known to have inhibitory effects on colon cancer [34] and protective effects on β-cells [35]. Previously, we found that BPCA shows anti-inflammatory effects by inhibiting ROS overproduction [36,37]. Additionally, protocatechualdehyde, which has a structure similar to that of BPCA, can be protective against mitochondrial dysfunction [38]. Together, these observations indicate the role of BPCA in regulating cellular redox balance. Therefore, in this study, we examined whether BPCA improves β-cell function and provides protection against PA toxicity.

## 2. Materials and Methods

### 2.1. Preparation of 5-Bromoprotocatechualdehyde (BPCA) from P. japonica

**Figure 1:**
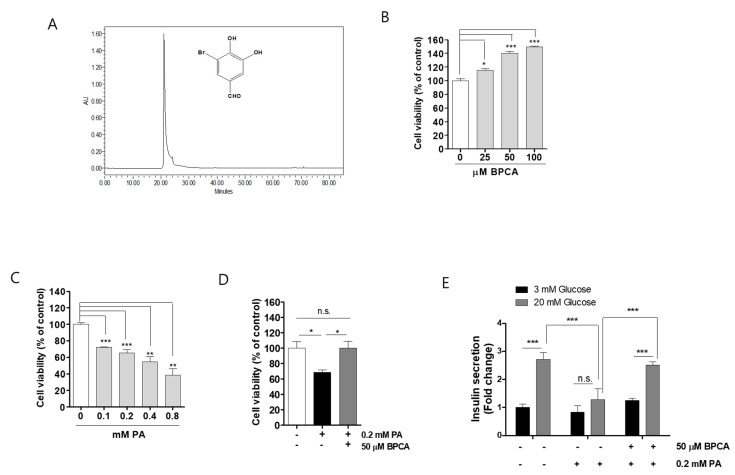
5-Bromoprotocatechualdehyde (BPCA) isolated from *Polysiphonia japonica* combats palmitate-induced toxicity and dysfunction in Ins-1 cells. (**A**). Chemical structure and high-performance liquid chromatography (HPLC) purity profile of BPCA. (**B**). Ins-1 cells were incubated with the indicated concentrations of BPCA (25, 50, and 100 µM) for 24 h. (**C**). Ins-1 cells were incubated with the indicated concentrations of palmitic acid (PA) (0.1, 0.2, 0.4, and 0.8 mM) for 24 h. (**D**). Ins-1 cells were incubated with 50 µM BPCA for 1 h and then further incubated with/without 0.2 mM PA for 24 h. CCK-8 assays were subsequently performed as described in “Materials and Methods”. (**E**). Ins-1 cells were incubated with 50 µM BPCA in 5 mM glucose media for 1 h and then further incubated with or without 0.2 mM PA for 24 h. Thereafter, the cells were starved in 0.2 mM glucose-containing Krebs–Ringer bicarbonate (KRB) buffer for 2 h. Insulin release was measured after 2 h of incubation in either 3 or 20 mM glucose. ELISA assays for insulin were subsequently performed. Data are expressed as the fold change from untreated cells in 3 mM glucose. * *p* < 0.05, ** *p* < 0.01, *** *p* < 0.001, n.s. = no significance.

The red seaweed *Polysiphonia japonica* (*P. japonica*) was collected along the coast of Jeju Island, Korea, between late fall 2015 and early spring 2016. After freezing the sample, it was lyophilized and then homogenized using a grinder prior to extraction. The diethyl ether fraction of the crude *P. japonica* extract was applied to silica gel (Sigma, St. Louis, MO, USA) and Sephadex LH-20 column (GE Healthcare, Chicago, IL, USA) chromatography (Supplementary Figure S1), and BPCA was purified through reversed-phase high-performance liquid chromatography (HPLC). The purity of BPCA was >96.5%, and its chemical structure is shown in Figure 1A.

### 2.2. Cell Culture

A rat pancreatic β-cell line—Ins-1 cells—was purchase from Merckmillipore (catalogue #.Scc-207, Darmstadt, Germany) and cultured in RPMI 1640 (Welgene, Kyungsangbuk-do, Korea) supplemented with 10% FBS (Gibco, Waltham, MA, USA), 100 U/mL penicillin, 100 μg/mL streptomycin (Welgene), and 55 µM β-mercaptoethanol (Gibco, Waltham, MA, USA) in a humidified incubator with 5% CO_2_.

### 2.3. Palmitic Acid Preparation

Stock solution of palmitic acid (PA; Sigma, St. Louis, MO, USA) was prepared by conjugating with fatty-acid-free BSA (bovine serum albumin; Sigma, St. Louis, MO, USA), as reported previously [39]. Briefly, palmitic acid was dissolved in 0.1 N NaOH (Daejung, Seoul, Korea) at 60 °C and diluted in a 1:10 ration in a prewarmed 12% BSA solution to obtain a final concentration of 10 mM. The control media were contained 0.1 N NaOH and BSA, but did not contain lipids.

### 2.4. Assessment of Cell Viability

Cell viability was determined using a cell-counting kit (CCK, D-Plus^TM^; Dongin LS, Kyunggi-do, Korea) that measured mitochondrial dehydrogenase activity. For cell counting, the Ins-1 cells (5 × 10^4^ cells/well) were seeded onto 96-well plates. After 16 h, the cells were treated with BPCA and/or PA. We treated the cells with different concentrations of BPCA (25, 50, and 100 µM) and PA (0.1, 0.2, 0.4, and 0.8 mM) for 24 h for toxicity assessment and chose the concentrations of 0.2 mM PA, which resulted in about 60% cell viability, and 50 µM of BPCA, which resulted in 140% cell viability, i.e., a proliferative effect without toxicity, for further study. To study the protective effects of BPCA, cells were pretreated with a vehicle (control, dimethylsulfoxide (DMSO)) or 50 µM BPCA for 1 h, and were subsequently incubated with or without 0.2 mM PA for 24 h at 37 °C. The CCK solution was then added to the wells to obtain a total reaction volume of 110 µL. After 1.5 h of incubation, the absorbance was measured at λ = 450 nm. The formazan generated in the control cells was used to 100% viability.

### 2.5. Measurement of Insulin Content

Ins-1 cells (10^5^ cells/well) were plated on 24-well plates, as described previously [40]. The cells were incubated in Krebs–Ringer bicarbonate (KRB) buffer with 3 or 20 mM glucose for 2 h at 37 °C. The supernatant was collected and used to measure the levels of insulin released using an ELISA kit according to manufacturer’s protocol (ALPCO, Salem, NH, USA). Insulin content was normalized to the protein content determined using a DC^TM^ protein assay kit (Bio-Rad, Hercules, CA, USA).

### 2.6. Measurement of Cell Death

Ins-1 cells (0.8 × 10^4^ cells/well) were seeded onto 24-well plates. The cells were treated with a vehicle (control, DMSO) or 50 µM BPCA, and 1 h later, 0.2 mM PA was added. After 24 h, 10 µg/mL of Hoechst 33342 (HO342, Sigma, St. Louis, MO, USA) or 50 µg/mL of propidium iodide (PI, Sigma, St. Louis, MO, USA) was added, and the cells were incubated for 10 min at 37 °C. Cell death (apoptosis or necrosis) induced by PA was determined through fluorescence microscopy (Zeiss, Oberkochen, Germany). The red–green–blue (RGB) image was analyzed for quantitative evaluation using the ImageJ software (NIH, Bethesda, MD, USA), and the mean value was presented in bar graphs.

### 2.7. Western Blotting

Ins-1 cells (4 × 10^5^ cells/well) were seeded onto six-well plates, and the cells were incubated with DMSO (control) or 50 µM BPCA for 1 h and further incubated with or without 0.2 mM PA for 24 h. The cells were lysed using RIPA buffer (GenDEPOT, Barker, TX, USA) with a protease inhibitor cocktail (GenDEPOT, Barker, TX, USA) for 20 min on ice. The lysates were centrifuged at 12,000 rpm for 20 min at 4 °C, and the supernatant was used for Western blotting. The protein concentrations were measured using a DC^TM^ protein assay kit (Bio-Rad, Hercules, CA, USA). The following step was performed as described in our previous study [35]. The antibodies used for this experiment were purchased from Cell Signaling Technology (CST, Danvers, MA, USA) for anti-Bcl-2, anti-Bax, anti-cleaved caspase-3, anti-cytochrome *C*, and anti-parkin; anti-β-actin was purchased from Santa Cruz Biotechnology (Santa Cruz, CA, USA).

### 2.8. Immunocytochemistry

Ins-1 cells cultured on coverslips were incubated with a vehicle (control, DMSO) or 50 µM BPCA for 1 h, after which 0.2 mM PA was added. After 24 h, the coverslips were washed twice with PBS and fixed in 4% paraformaldehyde for 15 min at room temperature. The fixed cells were washed with PBS, blocked with PBS containing 1% BSA and 0.1% Triton X-100 for 30 min at room temperature, and incubated overnight with anti-cytochrome *C* (Santa Cruz) and anti-Tom 20 (Santa Cruz) at 4 °C. Cells were then stained with fluorescence-conjugated secondary antibody (Life Technologies, Waltham, MA, USA) for 2 h, mounted using VECTASHIELD (Vector Laboratories, Burlingame, CA, USA), and observed under a confocal microscope (LSM700, Zeiss, Oberkochen, Germany). To evaluate cytochrome *C* and Tom 20, five random fields were selected in each experiment and 10–12 cells were imaged in each field. To quantitatively evaluate the fluorescent images, the RGB image was analyzed using the ImageJ software (NIH), and the mean value was shown in bar graphs.

### 2.9. Estimation of the Intracellular Reactive Oxygen Species (ROS) Levels

Ins-1 cells (1 × 10^5^ cells/well) were seeded onto 24-well plates. The cells were treated with a vehicle (control, DMSO) or 50 µM BPCA, and 1 h later, 0.2 mM PA was added, and the cells were incubated for 12 h. Intracellular ROS production was described previously [41]. Briefly, 5 μg/mL 2,7-dichlorofluorescein diacetate (DCFH-DA; Invitrogen, Carlsbad, CA, USA) was added, and cells were incubated for 30 min at 37 °C. The fluorescence image was observed using a fluorescence microscope (AXIO, Zeiss, Oberkochen, Germany). To quantitatively evaluate the fluorescent images, the RGB image was analyzed using the ImageJ software (NIH), and the mean value was shown in bar graphs.

### 2.10. Estimation of the Electron Spin Resonance (ESR) Spectrum

Ins-1 cells (4 × 10^5^ cells/well) were seeded onto six-well plates; the cells were incubated with a vehicle (control, DMSO) or 50 µM BPCA for 1 h, and then further incubated with or without 0.2 mM PA for 24 h. The cells were dissociated with trypsin, resuspended in PBS, and placed into a quartz tube using spin trap chemicals that can form stable radical adducts: 5-(diethoxyphosphoryl)-5-methyl-1-pyrroline N-oxide (DEPMPO; sigma, St. Louis, MO, USA) for the superoxide radical, 5,5-dimethyl-pyrrolidine-1-oxyl (DMPO) for the hydroxyl radical, and diethyldithiocarbamate and Fe-citrate for the nitric oxide radical. Intracellular radicals were respectively detected through electron spin resonance, and the results were recorded using a JES-FA ESR (electron spin resonance) spectrometer (JEOL Ltd. Tokyo, Japan) using the methods indicated in a previous study [42].

### 2.11. Treatment of Zebrafish Embryos with BPCA and PA

Transgenic zebrafish expressing enhanced green fluorescent protein under the control of the insulin promoter *Tg*(*ins*-egfp) were obtained from the Korean Zebrafish Organogenesis Mutant Bank and used in the experiment. Approximately 3 days post-fertilization (dpf) embryos (*n* = 10–12) were transferred to a 24-well plate and maintained in 1 mL of embryo media (0.003% sea salt, 0.0075% calcium sulfate). To determine the effect of BPCA, embryos were incubated in the presence of BPCA for 1 h prior to the addition of PA (0.2 mM) for 24 h. After that, the embryos were further incubated with basal glucose (3 mM) or stimulatory glucose (20 mM) for 3.5 h. Then, the embryos were rinsed in embryo media and anaesthetized with 2-phenoxy ethanol (Sigma, St. Louis, MO, USA). The embryos were washed twice with PBS and fixed in 4% paraformaldehyde overnight at 4 °C. The fixed embryos were then washed with PBS for 5 min at room temperature. After washing several times with PBS, the pancreata were isolated from the embryos, stained with DAPI (Invitrogen, Carlsbad, CA, USA) for 10 min, mounted on slides with Vectashield (Vector Laboratories, Burlingame, CA, USA), and observed with a confocal microscope (Zeiss, Oberkochen, Germany). The ImageJ software (NIH) was used to quantify the fluorescence and number of cells in the zebrafish, and the mean value was presented in a bar graph. Zebrafish embryos were handled in accordance with the guidelines of Gachon University.

### 2.12. Measurement of Heart Rates

The heart rates of both the atrium and ventricle were measured at 35 h post-fertilization (hpf) to determine the sample toxicity [43]. Counting and recording of atrial and ventricular contractions were performed for 30 s under a microscope, and the results were presented as the average heart rates per min.

### 2.13. Statistical Analysis

Significant differences were compared using one-way analysis with a subsequent multiple comparison test (Tukey) of variance using GraphPad prism version 6.0 (GraphPad software, San Diego, CA, USA), as well as one-way analysis of variance followed by Tukey’s post-hoc multiple comparison tests for the cell assay. The data are presented as means ± SEM (standard error of the mean). *P* values of less than 0.05 were considered to indicate statistical significance.

## 3. Results

### 3.1. BPCA Combats against Palmitate-Induced Toxicity and Maintains Insulin Secretion in Ins-1 Cells

In our previous study, we found that *P. japonica* extract (PJE) showed prominent protective effects for β-cells, and one of its target standards was BPCA [35]. Therefore, we isolated the target chemical compound 5-bromoprotocatechualdehyde (BPCA, Figure 1A) from JPE. First, to determine whether BPCA has a protective effect on palmitate-induced cytotoxicity, Ins-1 cells were treated with either BPCA or palmitate alone, or were preincubated with BPCA for 1 h and further incubated with palmitate for 24 h. BPCA alone did not show any cytotoxicity in Ins-1 cells in the concentration range tested (25–100 µM), but rather induced proliferation (Figure 1B). A significantly lower cell viability was observed in Ins-1 cells treated with palmitate in a dose-dependent manner (Figure 1C). Pretreatment with 50 µM BPCA increased the cell viability relative to the control in the presence of 0.2 mM palmitate (Figure 1D), suggesting that BPCA possesses a cytoprotective effect against palmitate-induced damage in Ins-1 cells. To investigate whether BPCA has protective effects on the palmitate-induced β-cell dysfunction, we measured the insulin secretion in BPCA-treated Ins-1 cells in the presence of palmitate. Although palmitate had no effect on basal insulin secretion (3 mM glucose), insulin secretion stimulated by high glucose concentration (20 mM) was inhibited by treatment with palmitate. When Ins-1 cells were preincubated with BPCA prior to palmitate treatment, the suppressed insulin secretion was restored to normal levels (Figure 1E), suggesting that the BPCA has protective effects against palmitate-induced inhibition of insulin secretion in Ins-1 cells.

### 3.2. BPCA Protects against Palmitate-Induced Cell Damage by Preventing ROS Overproduction and Promoting Antioxidant Activity in Ins-1 Cells

**Figure 2:**
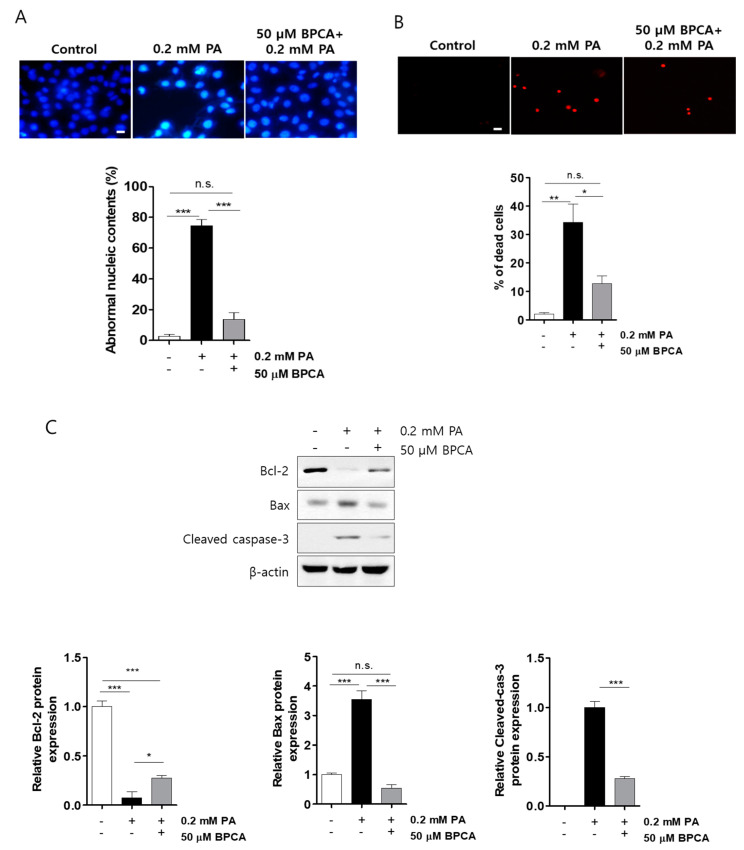
5-Bromoprotocatechualdehyde (BPCA) isolated from *Polysiphonia japonica* protects PA-induced cell death in Ins-1 cells. Ins-1 cells were incubated with 50 µM BPCA for 1 h and then further incubated with/without 0.2 mM PA for 24 h. (**A**) Further incubation with Hoechst 33342 (HO342, 10 µg/mL) subsequently performed in the images. (**B**) Further incubation with propidium iodide (PI, 50 µg/mL) subsequently performed in the images. Scale bar: 10 µm. (**C**). Western blotting was subsequently performed as described in “Materials and Methods”. * *p* < 0.05, ** *p* < 0.01, *** *p* < 0.001, n.s. = no significance.

To determine whether BPCA had a protective effect on palmitate-induced cell damage, the nuclei of Ins-1 cell were stained with either Hoechst 33342 (HO342) or propidium iodide (PI) to detect apoptosis or necrosis, respectively. Control cells had intact nuclei, whereas palmitate-treated cells showed nuclear fragmentation and damage, which are characteristics of apoptosis (Figure 2A) and necrosis (Figure 2B). However, the amount of fragmentation and damage caused by palmitate treatment was reduced in cells pretreated with BPCA (Figure 2A,B). Thus, to understand how BPCA protects against palmitate-induced damage in Ins-1 cells, we assessed the levels of intracellular proteins related to cellular damage, including Bcl-2, Bax, and cleaved-caspase-3. The results showed that palmitate increased the expression of pro-apoptotic protein Bax and induced caspase-3 cleavage. However, abundance of these proteins was significantly reduced in the BPCA-pretreated cells. In addition, expression of Bcl-2, the anti-apoptotic protein, was significantly lower in palmitate-treated cells, whereas the protein level was elevated by BPCA pretreatment (Figure 2C), suggesting that BPCA protects against palmitate-induced damage in Ins-1 cells.

**Figure 3:**
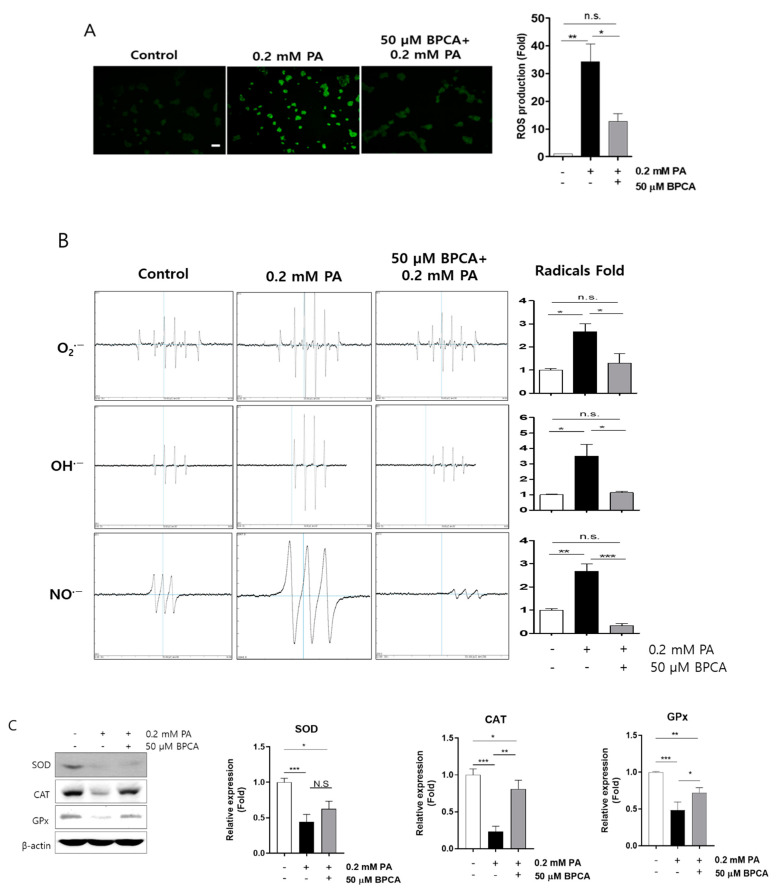
5-Bromoprotocatechualdehyde (BPCA) isolated from *Polysiphonia japonica* prevents PA-induced reactive oxygen species (ROS) overproduction by preserving antioxidant enzymes in Ins-1 cells. Ins-1 cells were incubated with 50 µM BPCA for 1 h, and then further incubated with/without 0.2 mM PA for 12 h. (**A**) Intracellular ROS was subsequently measured using 10 µM 2,7-dichlorofluorescein diacetate (DCFH-DA). Scale bar: 10 µm. (**B**) Radical production was subsequently determined using an electron spin resonance (ESR) spectrometer as described in “Materials and Methods”. (**C**) Western blotting was subsequently performed as described in “Materials and Methods”. * *p* < 0.05, ** *p* < 0.01, *** *p* < 0.001, n.s. = no significance.

The structure of BPCA resembles those of monomer polyphenolic compounds (Figure 1A), and it is well established that polyphenolic compounds are powerful free radical scavengers. In addition, it was found that the same chemical structure originated from same family seaweed inhibited ROS overproduction in kidney cells [37], and it showed UVB protection by enhancing the antioxidant system in keratinocyte [44]. Therefore, we determined the free radical scavenging capacity of BPCA in insulin-secreting β-cells. ROS overproduction was observed after palmitate treatment, whereas ROS levels were reduced with the 50 μM BPCA pretreatment before the 0.2 mM palmitate treatment (Figure 3A). To confirm the function of BPCA in scavenging palmitate-induced ROS, the cellular levels of free radicals, including superoxide, hydroxyl radicals, and nitric oxide radicals, were examined and found to be increased by palmitate treatment, but were largely restored to the control levels by BPCA pretreatment applied prior to 0.2 mM palmitate treatment (Figure 3B). Pancreatic β-cells are vulnerable to oxidative stress due to insufficient levels of antioxidant enzymes. Accordingly, we next determined the levels of endogenous antioxidant enzymes and found that they were reduced by palmitate treatment. Importantly, BPCA pretreatment provided protection against their reduction in cells treated with 0.2 mM palmitate (Figure 3C). Taken together, these results suggest that BPCA protects cells against palmitate-induced cell death by limiting ROS overproduction through the preservation of antioxidant enzymes.

### 3.3. BPCA Preserves Cytochrome C Release and Fragmentation of Mitochondria by Protecting Parkin Protein Degradation in Ins-1 Cells

**Figure 4:**
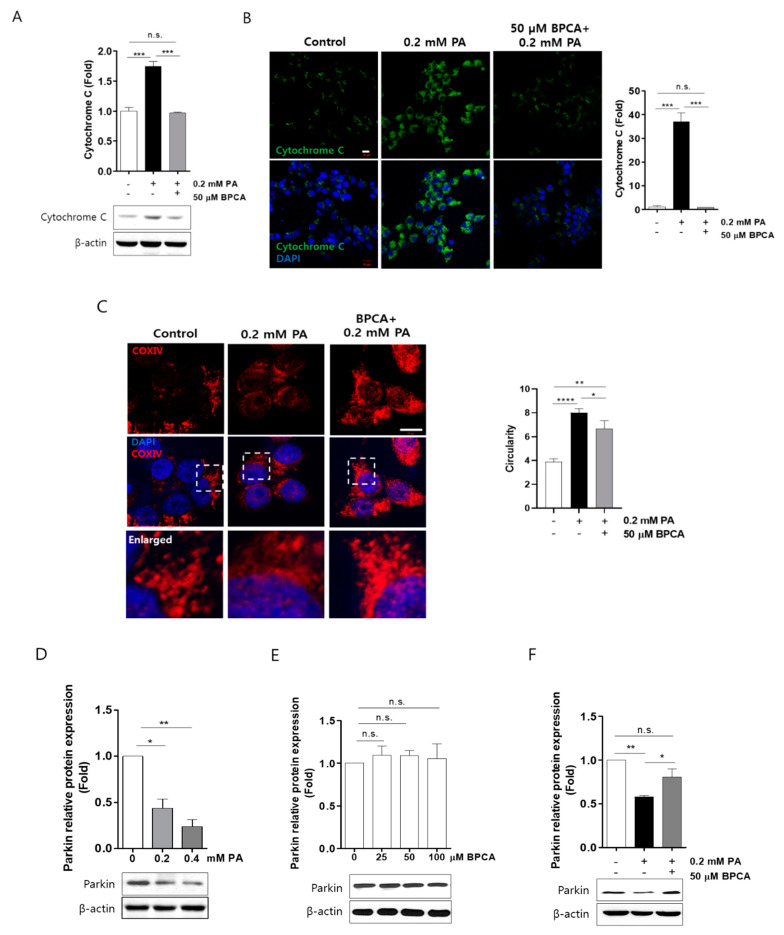
5-Bromoprotocatechualdehyde (BPCA) isolated from *Polysiphonia japonica* protects PA-induced degradation of parkin by combating cytochrome *C* release and morphology alteration of mitochondria in Ins-1 cells. Ins-1 cells were incubated with 50 µM BPCA for 1 h and then further incubated with/without 0.2 mM PA for 24 h. (**A**) Western blotting was subsequently performed as described in “Materials and Methods”. (**B**,**C**) Confocal image was subsequently performed as described in “Materials and Methods”. Scale bar: 10 µm. Circularity measures the average value per high-resolution micrograph using ImageJ. (**D**) Ins-1 cells were incubated with the indicated concentrations of PA (0.2 and 0.4 mM) for 24 h. (**E**) Ins-1 cells were incubated with the indicated concentrations of BPCA (25, 50, and 100 µM) for 24 h. (**F**) Ins-1 cells were incubated with 50 µM BPCA for 1 h and then further incubated with/without 0.2 mM PA for 24 h. Western blotting was subsequently performed as described in “Materials and Methods”. * *p* < 0.05, ** *p* < 0.01, *** *p*<0.005, **** *p*<0.001, n.s. = no significance.

Lipotoxicity is implicated in mitochondrial dysfunction and ROS overproduction, and mitochondria can initiate apoptosis through the release of cytochrome *C* [45]. Therefore, to investigate whether BPCA protects Ins-1 cells against mitochondrial dysfunction, we determined the effects of BPCA on cytochrome *C* expression in palmitate-treated cells. As expected, cytochrome *C* protein levels were increased by palmitate treatment, and this induction was reduced to the control levels by BPCA pretreatment in the presence of 0.2 mM palmitate (Figure 4A,B).

Parkin plays a crucial role in clearance of ROS and elimination of damaged mitochondria [46,47]. Parkin deficiency induces mitochondrial fragmentation and causes dysfunction of pancreatic β-cells [21]. We observed that the palmitate-induced mitochondrial fragmentation was blunted by BPCA pretreatment in cells exposed to 0.2 mM palmitate (Figure 4C), suggesting that BPCA prevents palmitate-induced mitochondrial fragmentation. Furthermore, we determined whether BPCA affects parkin expression in Ins-1 cells and found that palmitate treatment reduced parkin protein levels in a dose-dependent manner (Figure 4D). However, the expression of parkin protein was not altered by BPCA (25 μM, 50 μM, and 100 μM) treatment alone (Figure 4E). Interestingly, pretreatment with 50 μM BPCA preserved the expression of parkin protein in the presence of 0.2 mM palmitate (Figure 4F). These results suggest that BPCA prevents palmitate-induced degradation of parkin, thereby preserving mitochondrial function.

### 3.4. BPCA Protects against PA-Induced β-Cell Dysfunction in Zebrafish

**Figure 5:**
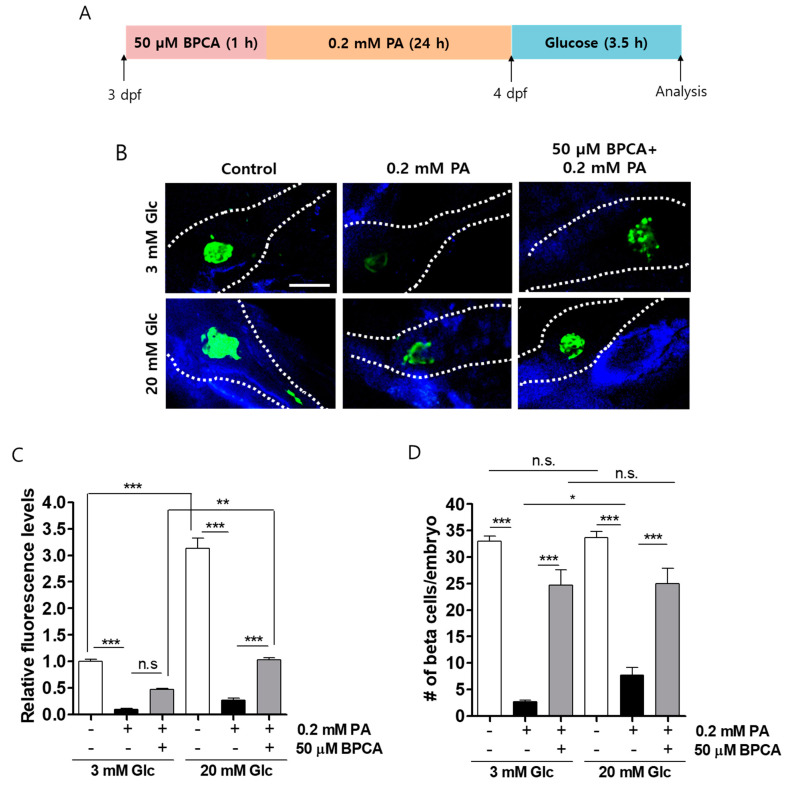
5-Bromoprotocatechualdehyde (BPCA) isolated from *Polysiphonia japonica* protects against PA-induced β-cell dysfunction in zebrafish. (**A**) Zebrafish were incubated with 50 µM BPCA and 0.2 mM PA from 3 to 4 days post-fertilization (dpf). BPCA was added 1 h prior to PA treatment. Thereafter, the zebrafish were incubated with 3 or 20 mM glucose for 3.5 h. (**B**) Confocal microscopy images of the pancreata of the zebrafish. Scale bar: 100 µm. (**C**). Relative EGFP fluorescence levels from (**B**). (**D**) Numbers of β-cells per embryo from (**B**). *n* = 4~6. * *p* < 0.05, ** *p* < 0.005, *** *p* < 0.001, n.s. = no significance.

Next, we determined whether BPCA has a protective effect on β-cells against palmitate in vivo. Zebrafish embryos were preincubated in 50 μM BPCA for 1 h, further incubated with 0.2 mM PA for 24 h, and stimulated with 3 or 20 mM glucose for 3.5 h (Figure 5A). EGFP (enhanced green fluorescence protein) expression in β-cells was reduced by palmitate treatment, whereas a higher expression of EGFP was observed in BPCA-pretreated embryos. Although palmitate treatment had no effect on basal insulin secretion (3 mM glucose), insulin secretion after stimulation with high glucose concentrations (20 mM) was significantly inhibited by treatment with palmitate, and insulin secretion was preserved by BPCA pretreatment in zebrafish embryos (Figure 5B,C), suggesting that BPCA protects against palmitate-induced defects in insulin secretion in zebrafish embryos. Similarly, the number of EGFP-positive (insulin-secreting) β-cells was reduced by treatment with 0.2 mM palmitate, and these numbers were preserved by 50 μM BPCA pretreatment in both conditions of 3 and 20 mM glucose (Figure 5D). Taken together, these results indicate that BPCA pretreatment protects palmitate-induced β-cell damage in vivo.

## 4. Discussion

Prolonged hyperglycemia is a causative factor for oxidative and mitochondrial stress in diabetic patients [48]. During this pathophysiology, glucose auto-oxidation and protein glycosylation result in the overproduction of free radicals. These free radicals are key players in the stress-mediated damage of various cells, including β-cells [49,50]. In the present study, we have provided, to the best of our knowledge, the first evidence regarding the potential role of BPCA in combating palmitate-induced adverse effects on pancreatic β-cells.

Most risk variants of T2D in healthy populations play a role by impairing insulin secretion, which leads to β-cell dysfunction, rather than insulin action resulting in insulin resistance, which indicates that the inherited abnormality in β-cell function or mass (or both) is an important precursor of T2D [51,52,53]. Therefore, the survival of pancreatic β-cells is critical for insulin secretion. Here, we provide evidence that BPCA prevents β-cell damage and dysfunction of insulin secretion in not only Ins-1 cells, but also in vivo in zebrafish embryos exposed to palmitate (Figure 1E and Figure 5).

Diabetes mellitus treatments are focused on improving insulin secretion. Despite treatment, diabetic patients need insulin injection therapy in the later stages of the disease, which is possibly due to loss of β-cells [54]. In this context, an attempt to preserve β-cells would be a promising strategy for preventing severe progression of DM. From another point of view, prolonged exposure to glucose, nutrients, or FFAs, especially the saturated FFAs, such as PA, can cause insulin-producing β-cell damage and dysfunction in insulin secretion [55,56,57,58]. To confirm this, our study applied different concentrations of PA to stimulate β-cells and established corresponding models. As observed in previous studies [59,60,61], our results showed that even lower concentrations of PA (0.2 mM) significantly induced cell necrosis and apoptosis in Ins-1 cells (Figure 2A,B). In addition, we found that a novel natural monomeric polyphenolic compound, BPCA, played a critical role in protecting β-cells from lipotoxicity and glucotoxicity (Figure 1, and rescued the decrease in PA-induced insulin secretion not only in Ins-1 cells, but in β-cells of zebrafish embryos as well (Figure 1E and Figure 5).

Impaired mitochondrial function is commonly observed in DM and metabolic syndromes [62] owing to β-cell mitochondria, which are fuel integrators of β-cells that generate signals for insulin secretion and regulate β-cell function [63,64]. The potential to improve β-cell function by reducing toxic mitochondrial ROS may prove to be a useful approach for mitochondrial-targeted therapy in the future [65,66]. Additionally, it may also be possible to maintain β-cell function by reducing the energy requirements of β-cells through a reduction in lipotoxicity [67]. In the present study, PA-induced β-cell apoptosis in the mitochondrial pathway was evaluated, and we provide evidence that BPCA upregulates anti-apoptotic Bcl-2 and downregulates pro-apoptotic Bax, cytochrome *C,* and cleaved-caspase-3 protein expression (Figure 2), suggesting that the combating effect of BPCA is associated with the mitochondrial pathway of PA-induced β-cell damage.

Aerobic cells are used as by-products to produce ROS, such as superoxide anions (O_2_^●−^), in the mitochondria [68,69]. Similarly, mitochondrial electron transport in β-cells is a major source of superoxide anions, which are reactive molecules, and can be converted into hydrogen peroxide (H_2_O_2_) by superoxide dismutase (SOD) isoenzymes, and then to oxygen and water by catalase (CAT), glutathione peroxidase (GPx), and peroxidase (Prx) [70]. β-cells possess lower amounts of antioxidant enzymes to fight against the sustained production of superoxide anions, which makes β-cells sensitive to ROS-related signals and oxidative and cytotoxic stress [71]. In mice, lipid infusion can reduce islet ROS, and treatment with a reducing agent or inhibition of NADPH oxidase can prevent β-cell dysfunction [72,73]. Nicotinamide protected Ins-1 cells against glucolipotoxicity due to its inhibitory activity on sirtuins [74]. Thus, the use of antioxidants or reducing agents will probably prevent oxidative stress and maintain β-cell function [75]. In this study, we found that BPCA serves as an inhibitor of excessively produced ROS and free radicals (O_2_^●−^, OH^●−^, and NO^●−^) under PA treatment (Figure 3A,B). In addition, BPCA enhanced the antioxidant enzymatic activities of SOD, CAT, and GPx against PA-treatment in β-cells (Figure 3C). These results suggested that BPCA can probably act as a reducing agent for PA-induced mitochondrial peroxidation.

Mitochondria are a source of ROS for detoxification; however, disruption of the delicate balance between ROS production and elimination might lead to the accumulation of ROS in the mitochondria. When the mitochondria eliminate ROS, a selective autophagy called mitophagy usually occurs, which is a key cellular mechanism that can be used to remove damaged mitochondria and to maintain the mitochondrial quality and function [76]. Parkin is involved in the regulation of autophagy [77]. In this process, the recruitment of parkin into the depolarized mitochondria triggers the damaged mitochondria that are isolated in the autophagosomes and transports them to lysosomes for degradation [78]. In Alzheimer’s disease (AD), a potential therapeutic strategy could be achieved through the upregulation of parkin-mediated mitophagy by overexpressing parkin to improve impaired mitochondria [76]. Furthermore, a protective effect of parkin in maintaining normal mitochondria in skeletal muscles has been described [78]. In the present study, PA-induced mitochondrial fragmentation was combated by BPCA pretreatment in the presence of 0.2 mM PA (Figure 4C), indicating that BPCA contributes to mitochondrial maintenance. Moreover, parkin protein expression was suppressed by PA treatment, which could be preserved by BPCA pre-treatment. These results suggest that BPCA might possess the ability to protect β-cells by maintaining the normal mitochondria or by upregulating the expression of parkin to preserve altered mitochondrial morphology.

Natural products have a long history of being used as an alternative treatment for diabetes, especially in Asia, India, and Africa [79,80,81,82,83,84]. Many studies have shown that natural products provide multiple anti-diabetic mechanisms, which preserve β-cell dysfunction (similar to the present study) through the inhibition of α-amylase and α-glucosidase [85,86,87] targeted signal pathways [88,89,90,91,92,93,94], as well as through the modulation of gut microbiota [95]. In the present study, we investigated the combating effects of BPCA isolated from *P. japonica* on β-cell dysfunction induced by PA. In fact, we found that BPCA possesses a high and specific combating effect on PA-induced Ins-1 cell dysfunction and improves β-cell function in zebrafish embryos. These results suggested that the maintenance of β-cell integrity by BPCA also contributes to the preservation of pancreatic β-cells.

## 5. Conclusions

We found that BPCA, a noble polyphenolic constituent, can effectively protect insulin-secreting β-cell toxicity induced by PA, which causes a release of ROS and leads to deficiency in the antioxidant enzyme, mitochondrial alteration, and parkin protein degradation. Moreover, BPCA improves β-cell function, including insulin secretion, in zebrafish embryo β-cells. Our results suggest that BPCA could be used as a novel preventive therapeutic agent for DM.

## Data Availability

All data generated or analyzed during this study are included in this published article.

## Supplementary Materials

The following are available online at https://www.mdpi.com/2076-3921/10/2/264/s1, Figure S1: Isolation scheme of BPCA.

## Abbreviations

AD	Alzheimer’s disease
ANOVA	analysis of variance
BPCA	5-bromoprotocatechualdehyde
BSA	bovine serum albumin
CAT	catalase
DCFH-DA	2,7-dichlorofluorescein diacetate
DEPMPO	5-(diethoxyphosphoryl)-5-methyl-1-pyrroline N-oxide
DM	diabetes mellitus
DMPO	5,5-dimethyl-pyrrolidine-1-oxyl
DMSO	dimethylsulfoxide
dpf	days post-fertilization
FBS	fetal bovine serum
FFAs	free fatty acids
GPx	glutathione peroxidase
GSIS	glucose-stimulated insulin secretion
HO342	Hoechst 33342
hpf	hours post-fertilization
HPLC	high performance liquid chromatography
KRB	Krebs–Ringer bicarbonate
PA	palmitate
PBS	phosphate buffer saline
PI	propidium iodide
*P. japonica*	*Polysiphonia japonica*
PJE	*P. japonica extract*
Prx	peroxidase
RNS	reactive nitrogen species
ROS	reactive oxygen species
SOD	superoxide dismutase
T1D	type 1 diabetes
T2D	type 2 diabetes

## References

[1] Ahmed N. (2005). Advanced glycation endproducts—Role in pathology of diabetic complications. Diabetes Res. Clin. Pract..

[2] Beisswenger P.J. (2014). Methylglyoxal in diabetes: Link to treatment, glycaemic control and biomarkers of complications. Biochem. Soc. Trans..

[3] Giacco F., Brownlee M. (2010). Oxidative stress and diabetic complications. Circ. Res..

[4] Fiorentino T.V., Prioletta A., Zuo P., Folli F. (2013). Hyperglycemia-induced oxidative stress and its role in diabetes mellitus related cardiovascular diseases. Curr. Pharm. Des..

[5] Chang K.C., Chung S.Y., Chong W.S., Suh J.S., Kim S.H., Noh H.K., Seong B.W., Ko H.J., Chun K.W. (1993). Possible superoxide radical-induced alteration of vascular reactivity in aortas from streptozotocin-treated rats. J. Pharmacol. Exp. Ther..

[6] Pieper G.M., Langenstroer P., Siebeneich W. (1997). Diabetic-induced endothelial dysfunction in rat aorta: Role of hydroxyl radicals. Cardiovasc. Res..

[7] Newbern D., Freemark M. (2011). Placental hormones and the control of maternal metabolism and fetal growth. Curr. Opin. Endocrinol. Diabetes Obes..

[8] Sachdeva M.M., Stoffers D.A. (2009). Minireview: Meeting the demand for insulin: Molecular mechanisms of adaptive postnatal beta-cell mass expansion. Mol. Endocrinol..

[9] Butler A.E., Janson J., Bonner-Weir S., Ritzel R., Rizza R.A., Butler P.C. (2003). Beta-cell deficit and increased beta-cell apoptosis in humans with type 2 diabetes. Diabetes.

[10] Tiwari B.K., Pandey K.B., Abidi A.B., Rizvi S.I. (2013). Markers of Oxidative Stress during Diabetes Mellitus. J. Biomark..

[11] Robertson R.P. (2004). Chronic oxidative stress as a central mechanism for glucose toxicity in pancreatic islet beta cells in diabetes. J. Biol. Chem..

[12] Li N., Frigerio F., Maechler P. (2008). The sensitivity of pancreatic beta-cells to mitochondrial injuries triggered by lipotoxicity and oxidative stress. Biochem. Soc. Trans..

[13] Gerber P.A., Rutter G.A. (2017). The Role of Oxidative Stress and Hypoxia in Pancreatic Beta-Cell Dysfunction in Diabetes Mellitus. Antioxid. Redox Signal..

[14] Supale S., Li N., Brun T., Maechler P. (2012). Mitochondrial dysfunction in pancreatic beta cells. Trends Endocrinol. Metab..

[15] Zhou Y.P., Pena J.C., Roe M.W., Mittal A., Levisetti M., Baldwin A.C., Pugh W., Ostrega D., Ahmed N., Bindokas V.P. (2000). Overexpression of Bcl-x_L_ in β-cells prevents cell death but impairs mitochondrial signal for insulin secretion. Am. J. Physiol. Endocrinol. Metab..

[16] Gurzov E.N., Eizirik D.L. (2011). Bcl-2 proteins in diabetes: Mitochondrial pathways of beta-cell death and dysfunction. Trends Cell Biol..

[17] Cnop M., Welsh N., Jonas J.-C., Jörns A., Lenzen S., Eizirik D.L. (2005). Mechanisms of pancreatic β-cell death in type 1 and type 2 diabetes: Many differences, few similarities. Diabetes.

[18] Grunnet L.G., Aikin R., Tonnesen M.F., Paraskevas S., Blaabjerg L., Storling J., Rosenberg L., Billestrup N., Maysinger D., Mandrup-Poulsen T. (2009). Proinflammatory cytokines activate the intrinsic apoptotic pathway in beta-cells. Diabetes.

[19] Gurzov E.N., Germano C.M., Cunha D.A., Ortis F., Vanderwinden J.M., Marchetti P., Zhang L., Eizirik D.L. (2010). p53 up-regulated modulator of apoptosis (PUMA) activation contributes to pancreatic beta-cell apoptosis induced by proinflammatory cytokines and endoplasmic reticulum stress. J. Biol. Chem..

[20] Cunha D.A., Ladriere L., Ortis F., Igoillo-Esteve M., Gurzov E.N., Lupi R., Marchetti P., Eizirik D.L., Cnop M. (2009). Glucagon-like peptide-1 agonists protect pancreatic beta-cells from lipotoxic endoplasmic reticulum stress through upregulation of BiP and JunB. Diabetes.

[21] Jin H.S., Kim J., Lee S.J., Kim K., Go M.J., Lee J.Y., Lee H.J., Song J., Jeon B.T., Roh G.S. (2014). The PARK2 gene is involved in the maintenance of pancreatic beta-cell functions related to insulin production and secretion. Mol. Cell. Endocrinol..

[22] Hoshino A., Ariyoshi M., Okawa Y., Kaimoto S., Uchihashi M., Fukai K., Iwai-Kanai E., Ikeda K., Ueyama T., Ogata T. (2014). Inhibition of p53 preserves Parkin-mediated mitophagy and pancreatic β-cell function in diabetes. Proc. Natl. Acad. Sci. USA.

[23] Ghorbani A. (2013). Best herbs for managing diabetes: A review of clinical studies. Braz. J. Pharm. Sci..

[24] Kim M.S., Kim J.Y., Choi W.H., Lee S.S. (2008). Effects of seaweed supplementation on blood glucose concentration, lipid profile, and antioxidant enzyme activities in patients with type 2 diabetes mellitus. Nutr. Res. Pract..

[25] Yang T.-H., Yao H.-T., Chiang M.-T. (2015). Red algae (*Gelidium amansii*) reduces adiposity via activation of lipolysis in rats with diabetes induced by streptozotocin-nicotinamide. J. Food Drug Anal..

[26] Kuda T., Tsunekawa M., Goto H., Araki Y. (2005). Antioxidant properties of four edible algae harvested in the Noto Peninsula, Japan. J. Food Compos. Anal..

[27] Ahn G.-N., Kim K.-N., Cha S.-H., Song C.-B., Lee J., Heo M.-S., Yeo I.-K., Lee N.-H., Jee Y.-H., Kim J.-S. (2007). Antioxidant activities of phlorotannins purified from Ecklonia cava on free radical scavenging using ESR and H_2_O_2_-mediated DNA damage. Eur. Food Res. Technol..

[28] Athukorala Y., Jeon Y.-J. (2005). Screening for angiotensin 1-converting enzyme inhibitory activity of Ecklonia cava. J. Food Sci. Nutr..

[29] Shibata T., Ishimaru K., Kawaguchi S., Yoshikawa H., Hama Y. (2008). Antioxidant activities of phlorotannins isolated from Japanese Laminariaceae. J. Appl. Phycol..

[30] Shim S.Y., Quang-To L., Lee S.H., Kim S.K. (2009). Ecklonia cava extract suppresses the high-affinity IgE receptor, FcepsilonRI expression. Food Chem. Toxicol..

[31] Jung H.A., Jin S.E., Ahn B.R., Lee C.M., Choi J.S. (2013). Anti-inflammatory activity of edible brown alga Eisenia bicyclis and its constituents fucosterol and phlorotannins in LPS-stimulated RAW264.7 macrophages. Food Chem. Toxicol..

[32] Santos S.A.O., Trindade S.S., Oliveira C.S.D., Parreira P., Rosa D., Duarte M.F., Ferreira I., Cruz M.T., Rego A.M., Abreu M.H. (2017). Lipophilic Fraction of Cultivated *Bifurcaria bifurcata* R. Ross: Detailed Composition and In Vitro Prospection of Current Challenging Bioactive Properties. Mar. Drugs.

[33] Rienks J., Barbaresko J., Oluwagbemigun K., Schmid M., Nothlings U. (2018). Polyphenol exposure and risk of type 2 diabetes: Dose-response meta-analyses and systematic review of prospective cohort studies. Am. J. Clin. Nutr..

[34] Gwak J., Park S., Cho M., Song T., Cha S.H., Kim D.E., Jeon Y.J., Shin J.G., Oh S. (2006). Polysiphonia japonica extract suppresses the Wnt/beta-catenin pathway in colon cancer cells by activation of NF-κB. Int. J. Mol. Med..

[35] Cha S.H., Kim H.S., Hwang Y., Jeon Y.J., Jun H.S. (2018). Polysiphonia japonica Extract Attenuates Palmitate-Induced Toxicity and Enhances Insulin Secretion in Pancreatic Beta-Cells. Oxid. Med. Cell. Longev..

[36] Ko E.-Y., Heo S.-J., Cho S.-H., Lee W., Kim S.-Y., Yang H.-W., Ahn G., Cha S.-H., Kwon S.-H., Jeong M.S. (2019). 3-Bromo-5-(ethoxymethyl)-1,2-benzenediol inhibits LPS-induced pro-inflammatory responses by preventing ROS production and downregulating NF-κB in vitro and in a zebrafish model. Int. Immunopharmacol..

[37] Cho S.H., Heo S.J., Yang H.W., Ko E.Y., Jung M.S., Cha S.H., Ahn G., Jeon Y.J., Kim K.N. (2019). Protective Effect of 3-Bromo-4,5-Dihydroxybenzaldehyde from Polysiphonia morrowii Harvey against Hydrogen Peroxide-Induced Oxidative Stress In Vitro and In Vivo. J. Microbiol. Biotechnol..

[38] Wang Y.H., Han Y.P., Yu H.T., Pu X.P., Du G.H. (2014). Protocatechualdehyde prevents methylglyoxal-induced mitochondrial dysfunction and AGEs-RAGE axis activation in human lens epithelial cells. Eur. J. Pharmacol..

[39] Sinha S., Perdomo G., Brown N.F., O’Doherty R.M. (2004). Fatty acid-induced insulin resistance in L6 myotubes is prevented by inhibition of activation and nuclear localization of nuclear factor kappa B. J. Biol. Chem..

[40] Oh Y.S., Lee Y.J., Park E.Y., Jun H.S. (2011). Interleukin-6 treatment induces beta-cell apoptosis via STAT-3-mediated nitric oxide production. Diabetes Metab. Res. Rev..

[41] Cha S.-H., Heo S.-J., Jeon Y.-J., Park S.M. (2016). Dieckol, an edible seaweed polyphenol, retards rotenone-induced neurotoxicity and α-synuclein aggregation in human dopaminergic neuronal cells. RSC Adv..

[42] Cha S.H., Han E.J., Ahn G., Jun H.S. (2019). Taurine-Rich-Containing Hot Water Extract of Loliolus Beka Gray Meat Scavenges Palmitate-Induced Free Radicals and Protects Against DNA Damage in Insulin Secreting β-Cells. Adv. Exp. Med. Biol..

[43] Cha S.-H., Lee J.-H., Kim E.-A., Shin C.H., Jun H.-S., Jeon Y.-J. (2017). Phloroglucinol accelerates the regeneration of liver damaged by H_2_O_2_ or MNZ treatment in zebrafish. RSC Adv..

[44] Piao M.J., Kang H.K., Yoo E.S., Koh Y.S., Kim D.S., Lee N.H., Hyun J.W. (2012). Photo-protective effect of *Polysiphonia morrowii* Harvey against ultraviolet B radiation-induced keratinocyte damage. J. Korean Soc. Appl. Biol. Chem..

[45] Kuzmicic J., Parra V., Verdejo H.E., Lopez-Crisosto C., Chiong M., Garcia L., Jensen M.D., Bernlohr D.A., Castro P.F., Lavandero S. (2014). Trimetazidine prevents palmitate-induced mitochondrial fission and dysfunction in cultured cardiomyocytes. Biochem. Pharmacol..

[46] Narendra D., Tanaka A., Suen D.-F., Youle R.J. (2008). Parkin is recruited selectively to impaired mitochondria and promotes their autophagy. J. Cell Biol..

[47] Ansari M.Y., Khan N.M., Ahmad I., Haqqi T.M. (2018). Parkin clearance of dysfunctional mitochondria regulates ROS levels and increases survival of human chondrocytes. Osteoarthr. Cartil..

[48] Tangvarasittichai S. (2015). Oxidative stress, insulin resistance, dyslipidemia and type 2 diabetes mellitus. World J. Diabetes.

[49] Uttara B., Singh A.V., Zamboni P., Mahajan R.T. (2009). Oxidative stress and neurodegenerative diseases: A review of upstream and downstream antioxidant therapeutic options. Curr. Neuropharmacol..

[50] Asmat U., Abad K., Ismail K. (2016). Diabetes mellitus and oxidative stress—A concise review. Saudi Pharm. J..

[51] Florez J.C. (2008). Newly identified loci highlight beta cell dysfunction as a key cause of type 2 diabetes: Where are the insulin resistance genes?. Diabetologia.

[52] Voight B.F., Scott L.J., Steinthorsdottir V., Morris A.P., Dina C., Welch R.P., Zeggini E., Huth C., Aulchenko Y.S., Thorleifsson G. (2010). Twelve type 2 diabetes susceptibility loci identified through large-scale association analysis. Nat. Genet..

[53] Petrie J.R., Pearson E.R., Sutherland C. (2011). Implications of genome wide association studies for the understanding of type 2 diabetes pathophysiology. Biochem. Pharmacol..

[54] Prentki M., Nolan C.J. (2006). Islet β cell failure in type 2 diabetes. J. Clin. Investig..

[55] Leonardi O., Mints G., Hussain M.A. (2003). Beta-cell apoptosis in the pathogenesis of human type 2 diabetes mellitus. Eur. J. Endocrinol..

[56] Shi G., Sun C., Gu W., Yang M., Zhang X., Zhai N., Lu Y., Zhang Z., Shou P., Zhang Z. (2014). Free fatty acid receptor 2, a candidate target for type 1 diabetes, induces cell apoptosis through ERK signaling. J. Mol. Endocrinol..

[57] Lupi R., Dotta F., Marselli L., Del Guerra S., Masini M., Santangelo C., Patane G., Boggi U., Piro S., Anello M. (2002). Prolonged exposure to free fatty acids has cytostatic and pro-apoptotic effects on human pancreatic islets: Evidence that beta-cell death is caspase mediated, partially dependent on ceramide pathway, and Bcl-2 regulated. Diabetes.

[58] Maedler K., Spinas G.A., Dyntar D., Moritz W., Kaiser N., Donath M.Y. (2001). Distinct effects of saturated and monounsaturated fatty acids on beta-cell turnover and function. Diabetes.

[59] El-Assaad W., Buteau J., Peyot M.L., Nolan C., Roduit R., Hardy S., Joly E., Dbaibo G., Rosenberg L., Prentki M. (2003). Saturated fatty acids synergize with elevated glucose to cause pancreatic beta-cell death. Endocrinology.

[60] Wehinger S., Ortiz R., Diaz M.I., Aguirre A., Valenzuela M., Llanos P., Mc Master C., Leyton L., Quest A.F. (2015). Phosphorylation of caveolin-1 on tyrosine-14 induced by ROS enhances palmitate-induced death of beta-pancreatic cells. Biochim. Biophys. Acta.

[61] Hao F., Kang J., Cao Y., Fan S., Yang H., An Y., Pan Y., Tie L., Li X. (2015). Curcumin attenuates palmitate-induced apoptosis in MIN6 pancreatic beta-cells through PI3K/Akt/FoxO1 and mitochondrial survival pathways. Apoptosis.

[62] Nisoli E., Clementi E., Carruba M.O., Moncada S. (2007). Defective mitochondrial biogenesis: A hallmark of the high cardiovascular risk in the metabolic syndrome?. Circ. Res..

[63] Wikstrom J.D., Sereda S.B., Stiles L., Elorza A., Allister E.M., Neilson A., Ferrick D.A., Wheeler M.B., Shirihai O.S. (2012). A novel high-throughput assay for islet respiration reveals uncoupling of rodent and human islets. PLoS ONE.

[64] Cerf M.E. (2013). Beta cell dysfunction and insulin resistance. Front. Endocrinol..

[65] Mahadevan J., Parazzoli S., Oseid E., Hertzel A.V., Bernlohr D.A., Vallerie S.N., Liu C.Q., Lopez M., Harmon J.S., Robertson R.P. (2013). Ebselen treatment prevents islet apoptosis, maintains intranuclear Pdx-1 and MafA levels, and preserves beta-cell mass and function in ZDF rats. Diabetes.

[66] Zhang C.Y., Parton L.E., Ye C.P., Krauss S., Shen R., Lin C.T., Porco J.A., Lowell B.B. (2006). Genipin inhibits UCP2-mediated proton leak and acutely reverses obesity- and high glucose-induced beta cell dysfunction in isolated pancreatic islets. Cell Metab..

[67] Kaufman B.A., Li C., Soleimanpour S.A. (2015). Mitochondrial regulation of beta-cell function: Maintaining the momentum for insulin release. Mol. Asp. Med..

[68] Forman H.J., Torres M. (2002). Reactive oxygen species and cell signaling: Respiratory burst in macrophage signaling. Am. J. Respir. Crit. Care Med..

[69] Zhang W., Feng D., Li Y., Iida K., McGrath B., Cavener D.R. (2006). PERK EIF2AK3 control of pancreatic β cell differentiation and proliferation is required for postnatal glucose homeostasis. Cell Metab..

[70] Russell E.G., Cotter T.G. (2015). New Insight into the Role of Reactive Oxygen Species (ROS) in Cellular Signal-Transduction Processes. Int. Rev. Cell Mol. Biol..

[71] Wang J., Wang H. (2017). Oxidative Stress in Pancreatic Beta Cell Regeneration. Oxid. Med. Cell. Longev..

[72] Koulajian K., Ivovic A., Ye K., Desai T., Shah A., Fantus I.G., Ran Q., Giacca A. (2013). Overexpression of glutathione peroxidase 4 prevents beta-cell dysfunction induced by prolonged elevation of lipids in vivo. Am. J. Physiol. Endocrinol. Metab..

[73] Koulajian K., Desai T., Liu G.C., Ivovic A., Patterson J.N., Tang C., El-Benna J., Joseph J.W., Scholey J.W., Giacca A. (2013). NADPH oxidase inhibition prevents beta cell dysfunction induced by prolonged elevation of oleate in rodents. Diabetologia.

[74] Lee S.J., Choi S.E., Jung I.R., Lee K.W., Kang Y. (2013). Protective effect of nicotinamide on high glucose/palmitate-induced glucolipotoxicity to INS-1 beta cells is attributed to its inhibitory activity to sirtuins. Arch. Biochem. Biophys..

[75] Sharma R.B., Alonso L.C. (2014). Lipotoxicity in the pancreatic beta cell: Not just survival and function, but proliferation as well?. Curr. Diabetes Rep..

[76] Wang H., Zhang T., Ge X., Chen J., Zhao Y., Fu J. (2020). Parkin overexpression attenuates Aβ-induced mitochondrial dysfunction in HEK293 cells by restoring impaired mitophagy. Life Sci..

[77] Drummond M.J., Addison O., Brunker L., Hopkins P.N., McClain D.A., LaStayo P.C., Marcus R.L. (2014). Downregulation of E3 ubiquitin ligases and mitophagy-related genes in skeletal muscle of physically inactive, frail older women: A cross-sectional comparison. J. Gerontol. A Biol. Sci. Med. Sci..

[78] Gouspillou G., Godin R., Piquereau J., Picard M., Mofarrahi M., Mathew J., Purves-Smith F.M., Sgarioto N., Hepple R.T., Burelle Y. (2018). Protective role of Parkin in skeletal muscle contractile and mitochondrial function. J. Physiol..

[79] Grosso G., Stepaniak U., Micek A., Kozela M., Stefler D., Bobak M., Pajak A. (2017). Dietary polyphenol intake and risk of type 2 diabetes in the Polish arm of the Health, Alcohol and Psychosocial factors in Eastern Europe (HAPIEE) study. Br. J. Nutr..

[80] Heo S.J., Hwang J.Y., Choi J.I., Han J.S., Kim H.J., Jeon Y.J. (2009). Diphlorethohydroxycarmalol isolated from *Ishige okamurae*, a brown algae, a potent alpha-glucosidase and alpha-amylase inhibitor, alleviates postprandial hyperglycemia in diabetic mice. Eur. J. Pharmacol..

[81] Rios J.L., Francini F., Schinella G.R. (2015). Natural Products for the Treatment of Type 2 Diabetes Mellitus. Planta Med..

[82] Salimifar M., Fatehi-Hassanabad Z., Fatehi M. (2013). A review on natural products for controlling type 2 diabetes with an emphasis on their mechanisms of actions. Curr. Diabetes Rev..

[83] Xiao J.B., Hogger P. (2015). Dietary polyphenols and type 2 diabetes: Current insights and future perspectives. Curr. Med. Chem..

[84] Shapiro K., Gong W.C. (2002). Natural products used for diabetes. J. Am. Pharm. Assoc. (1996).

[85] Apostolidis E., Lee C.M. (2010). In vitro potential of *Ascophyllum nodosum* phenolic antioxidant-mediated α-glucosidase and α-amylase inhibition. J. Food Sci..

[86] Nguyen D.V., Shaw L.C., Grant M.B. (2012). Inflammation in the pathogenesis of microvascular complications in diabetes. Front. Endocrinol..

[87] Hasnain S.Z., Prins J.B., McGuckin M.A. (2016). Oxidative and endoplasmic reticulum stress in beta-cell dysfunction in diabetes. J. Mol. Endocrinol..

[88] Kurimoto Y., Shibayama Y., Inoue S., Soga M., Takikawa M., Ito C., Nanba F., Yoshida T., Yamashita Y., Ashida H. (2013). Black soybean seed coat extract ameliorates hyperglycemia and insulin sensitivity via the activation of AMP-activated protein kinase in diabetic mice. J. Agric. Food Chem..

[89] Li S., Chen H., Wang J., Wang X., Hu B., Lv F. (2015). Involvement of the PI3K/Akt signal pathway in the hypoglycemic effects of tea polysaccharides on diabetic mice. Int. J. Biol. Macromol..

[90] Rosner M., Hanneder M., Siegel N., Valli A., Fuchs C., Hengstschlager M. (2008). The mTOR pathway and its role in human genetic diseases. Mutat. Res..

[91] Gurzov E.N., Stanley W.J., Pappas E.G., Thomas H.E., Gough D.J. (2016). The JAK/STAT pathway in obesity and diabetes. FEBS J..

[92] Chiang Y.T., Ip W., Jin T. (2012). The role of the Wnt signaling pathway in incretin hormone production and function. Front. Physiol..

[93] Liu M., Qin J., Hao Y., Liu M., Luo J., Luo T., Wei L. (2013). Astragalus polysaccharide suppresses skeletal muscle myostatin expression in diabetes: Involvement of ROS-ERK and NF-κB pathways. Oxid. Med. Cell. Longev..

[94] Siddle K. (2011). Signalling by insulin and IGF receptors: Supporting acts and new players. J. Mol. Endocrinol..

[95] Xu J., Lian F., Zhao L., Zhao Y., Chen X., Zhang X., Guo Y., Zhang C., Zhou Q., Xue Z. (2015). Structural modulation of gut microbiota during alleviation of type 2 diabetes with a Chinese herbal formula. ISME J..

