# Supplementary material for: 5-Bromoprotocatechualdehyde Combats against Palmitate Toxicity by Inhibiting Parkin Degradation and Reducing ROS-Induced Mitochondrial Damage in Pancreatic β-Cells
Source: Antioxidants (Basel). 2021 Feb 9;10(2):264. doi: 10.3390/antiox10020264 (PMC7914851; doi:10.3390/antiox10020264)
Supplement: Supplementary file 1 [file antioxidants-10-00264-s001.pdf]

Supplementary Figure S1. Isolation scheme of BPCA.

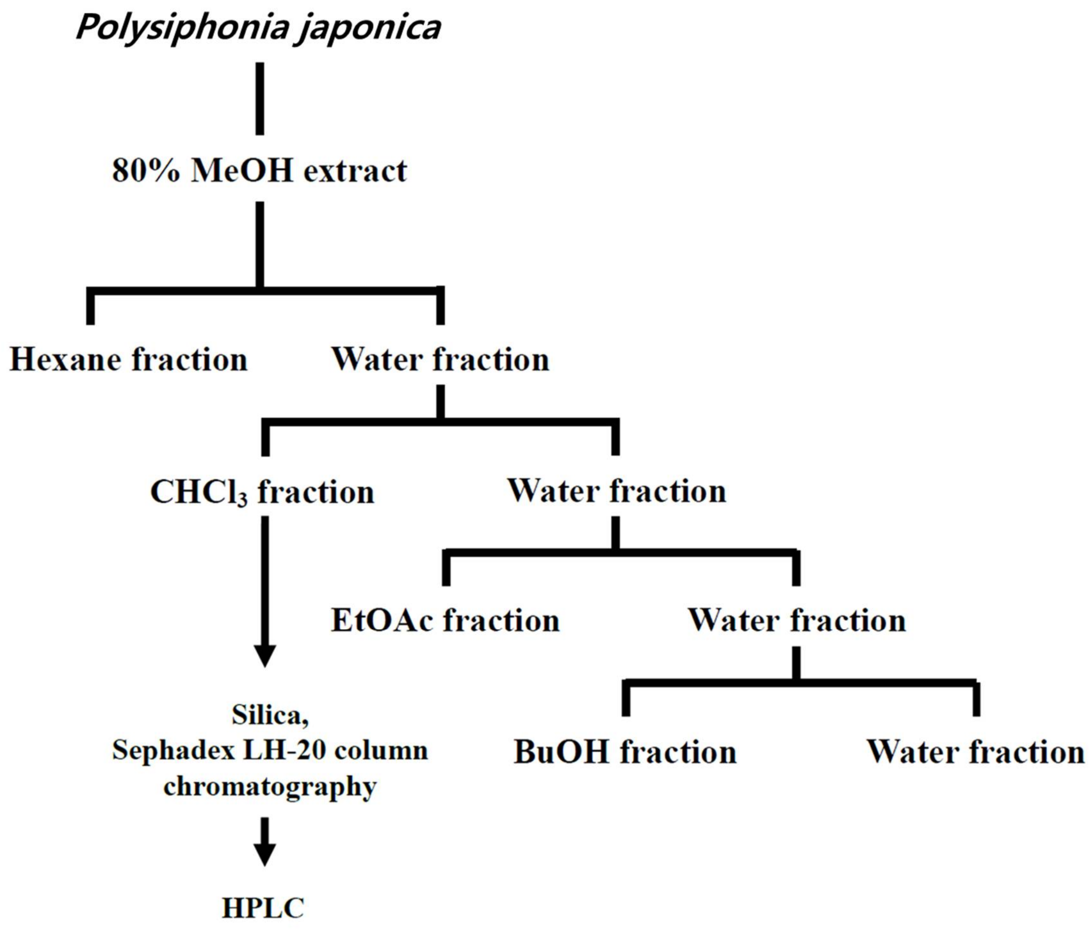

The dried *P. japonica* powder was extracted thrice with 80% aqueous methanol at room temperature. The liquid layer was obtained via filtration, and the filtrate was concentrated using an evaporator under reduced pressure. The extract was suspended in water, and the aqueous layer was partitioned with chloroform. Then, the chloroform fraction was fractionated by silica column chromatography with stepwise elution with a chloroform-methanol mixture (30:1→1:1) to separate the active fractions in the chloroform extract. A combined active fraction was further subjected to a Sephadex LH-20 column saturated with 100% methanol and then purified by reversed phase high-performance liquid chromatography (HPLC) using a Waters HPLC system (Alliance 2690; Waters Corp., Milford, MA, USA) equipped with a Waters 996 photodiode array detector and C18 column (J'sphere ODS-H80, 250 × 4.6 mm, 4 μm; YMC Co., Kyoto, Japan) by stepwise elution with a methanol-water gradient (UV range, 290 nm; flow rate, 1 ml/min).
